# Ethnic Disparities and Obesity Risk Factors in Pregnant Women: A Retrospective Observational Cohort Study

**DOI:** 10.3390/nu15040926

**Published:** 2023-02-12

**Authors:** Míriam Orós, Júlia Siscart, Daniel Perejón, Maria Catalina Serna, Pere Godoy, Blanca Salinas-Roca

**Affiliations:** 1 Family Medicine Department, University of Lleida, PC 25003 Lleida, Spain; 2 Primary Care Research Institute IDIAP Jordi Gol, Catalan Institute of Health, CP 08007 Barcelona, Spain; 3 Therapeutic Research Group in Primary Care (GRETAP), Catalan Institute of Health, CP 25007 Lleida, Spain; 4 Cambrils Health Center, CP 43850 Cambrils, Spain; 5 Serós Health Center, Catalan Institute of Health, PC 25183 Lleida, Spain; 6 Cervera Health Center, Catalan Institute of Health, PC 25200 Lleida, Spain; 7 School of Medicine, Lleida University, PC 25003 Lleida, Spain; 8 Eixample Health Center, Catalan Institute of Health, PC 25006 Lleida, Spain; 9 Institut de Recerca Biomédica (IRBLleida), PC 25198 Lleida, Spain; 10 CIBER of Epidemiology and Public Health (CIBERESP), Instituto Carlos III, PC 28005 Madrid, Spain; 11 Department of Nursing and Physiotherapy, University of Lleida, Montserrat Roig 2, PC 25198 Lleida, Spain; 12 Grow-Global Research on Wellbeing (GRoW) Research Group, Blanquerna School of Health Science, Ramon Llull University, Padilla, 326-332, PC 08025 Barcelona, Spain

**Keywords:** pregnancy, overweight, obesity, ethnicity, prevalence

## Abstract

(1) Background: This article focuses on the prevalence of overweight and obesity in pregnancy in different ethnic groups and assesses the existence of associated comorbidities. (2) Materials and Methods: A retrospective observational cohort study of 16803 pregnant women was carried out between 2012 and 2018 in the health region of Lleida (72% of the total). The relationship between overweight and obesity and different variables was analyzed by calculating the adjusted odds ratio (aOR) and 95% confidence intervals with multivariate logistic regression models. (3) Results: The prevalence of obesity in pregnant women rose from 11.1% in 2012 to 13.4% in 2018, and there was an age-related weight gain. A high incidence of overweight and obesity was recorded in pregnant women from ethnic groups: Maghrebi, sub-Saharan African and Latin America populations presented ORs of 4.08, 3.18 and 1.59, respectively. Hypertension was the variable most affected by body mass index (BMI) > 25 (OR = 3.39) followed by gestational diabetes mellitus (OR = 2.35). Depression was also associated with obesity. (4) Conclusions: The BMI of pregnant women is influenced by individual, ethnic and clinical factors. Mental health conditions such as depression are associated with BMI.

## 1. Introduction

Obesity continues to be a major public health concern. Overweight and obesity, defined as a body mass index (BMI) of 25 to < 30 or ≥ 30, have reached epidemic proportions in recent decades [1].

As part of this trend, the prevalence of overweight and obesity has risen in pregnant women. This increase is linked to a variety of complications that are considered the most common health risks during pregnancy [2,3]: gestational hypertension, preeclampsia, preterm birth, gestational diabetes mellitus (GDM), both small and large offspring, depression, instrumental or caesarean birth, and a higher prevalence of still birth and congenital defects, such as neurocognitive disorders [4,5,6].

During a healthy pregnancy, the maternal physiology changes to support the growth of the fetus. Thus, metabolic adjustments in maternal insulin sensitivity occur, depending on the demands of the specific stage of pregnancy. In early pregnancy, there is an increase in insulin sensitivity that promotes the uptake of glucose in the adipose tissue and prepares the organism for increased energy requirements that occur later. After this, the maternal metabolism switches to a state of relative insulin resistance, resulting in a modest increase in maternal blood glucose [3]. Observational evidence suggests that metabolic changes caused by maternal overweight and obesity affect epigenetic markers and influence offspring [7].

Previous studies have suggested that weight gain in pregnant women is associated with various factors beyond individual ones. For example, family and social factors are ascribed a potential influence on weight gain [8]. In this context, ethnicity may have an impact on the lifestyle and dietary habits that lead to increases in the BMI in pre-conceptional women.

Epidemiologic and prospective cohort studies have identified maternal and gestational conditions that increase the risk of obesity in children and of subsequent cardiometabolic disorders. It has been proposed that perinatal conditions raise the risk of many diseases later in life and specifically enhance the propensity for obesity [2]. In Europe, it is possible to distinguish a gradient in the prevalence of obesity in children from 10% in the north of Europe to up to 40% in the south [9]. The associated risks include a wide range of biological and environmental factors. In our view, the currently available data concerning maternal obesity and health and its associated social risk factors are insufficient. Despite the growing interest among the scientific community in the associations between maternal health status and childhood disease predisposition, the risk factors and mechanisms remain poorly defined. In this study, we analyzed the prevalence of overweight and obesity in pregnancy in different ethnic groups and in relation to the existence of associated comorbidity.

## 2. Materials and Methods

### 2.1. Design of the Study and Data Collection

This retrospective cohort study conducted in Lleida (Spain), assessed data from all pregnant women who gave birth in Hospital Arnau de Vilanova (Lleida, Spain) from 1 January 2012 to 31 December 2018.

Data were obtained from an internal data base CMBD (Group of Database) of the Catalan Health service and E-CAP from the Catalan Health Institute, corresponding to all eligible patients assigned to a primary care unit.

This article is part of the Iler Pregnancy project, a retrospective cohort study conducted in Lleida with the aim of evaluating the prevalence of chronic pathologies in pregnancy (hypothyroidism, depression, diabetes mellitus and obesity) and the therapeutic adherence to prescribed drugs [10].

### 2.2. Participants Eligibility Criteria

As inclusion criteria, women who had given birth between 1 January 2012 and 31 December 2018 were studied. Pregnancy data from the date of the last period to the date of delivery were included, so data from 2011 were reviewed for those pregnant women with a delivery date in 2012 but the date of their last period in 2011. Pregnant women who did not belong to the health region of Lleida were excluded. To evaluate the representativeness of the sample, the percentage of births studied (births registered at the Arnau de Vilanova University Hospital in Lleida) was calculated with respect to the total number of births in the health region of Lleida according to the data obtained from the database of the “Institute of Statistics of Catalonia” (IDESCAT) (Table 1).

### 2.3. Health Outcomes

In the pregnant women, the primary outcomes were the values for body mass index (BMI), which is routinely assessed during prenatal care. According to WHO recommendations, it is defined as weight for a healthy status [11]. Major adult BMI classifications are underweight (under 18.5 kg/m^2^), normal weight (18.5 to 24.9), overweight (25 to 29.9), and obese (30 or more). In the present study, BMI was calculated based on the prior weight to pregnancy.

Secondary outcomes in pregnant women were as follows: obstetrical and medical complications, such as number of pregnancies and twin pregnancies; abortions; prolonged or pre-term delivery; cesarean section; diabetes mellitus (code O24.9 at CIE-10.); arterial hypertension (code I10-I16 at l’ICD-10); dyslipidemia (code E78 at l’ICD-10); depression (codes F32.0-F32.9, F33.0-F33.3, F33.8, F33.9, F34.1, or F41.2 at l’ICD-10); hypothyroidism (code E03.9 and E02 of the ICD-10); preeclampsia (code O14.90 at ICD-10); and risk during pregnancy.

### 2.4. Statistical Analysis

A descriptive analysis was conducted. Numerical variables are described by mean and standard deviation and categorical variables by absolute and relative frequencies. Differences between groups were evaluated using Student’s t-test or Chi-square test, depending on whether the variables were numerical or categorical, respectively. Odds ratio was calculated, the relationship with other variables was analyzed using linear regression coefficients, and 95% confidence intervals were used.

### 2.5. Ethical Aspects

This study was approved by the Clinical Research Ethics Committee (CEIC) of the IDIAP Jordi Gol Research Institute under Code 19/194-P and carried out following the principles of the Declaration of Helsinki. SER information was obtained through centralized medical files in the ECAP database and extracted by the Department of Health Research Management and Evaluation.

Therefore, it was not necessary to request informed consent from the participants. The variables in the ECAP database were anonymously processed and with all the guarantees of confidentiality established by the National Law and Regulation 2016/679 of the European Parliament and of the Council on the protection of natural persons with regard to the use of personal information and the written circulation of this information.

## 3. Results

Of the 23,850 births in Hospital Arnau de Vilanova of Lleida (Spain), between the years 2012 to 2018, the sample included 16,803 pregnant women who met inclusion criteria. The prevalence of obesity in pregnant women ranged from 11.1% in 2012 to 13,4% in 2018. This trend was observed in overweight data for pregnant women, increasing from 20.8% (2012) to 22.8% (2018) (Table 2). Women who were pregnant from 2012 to 2018 of a normal weight were considered the majority population in this study (N = 11117). The prevalence was 25% for overweight and 15% for obesity. In all obese patients, we observed that 93% had a BMI between 30 and 40, 6.7% between 41 and 50; and only 3% had a BMI higher than 50.

Table 3 shows the maternal individual factors for the total sample. The appearance of overweight and obesity was significantly affected by maternal age. Pregnant women with overweight were typically over 35 years old (38.1%), whereas 30.9% was below 30 years, similar to those aged 30–35 years (31%). The majority of women were experiencing their first pregnancy. The results indicate a significant link between obesity and number of pregnancies, preeclampsia, caesarean and risk factors during pregnancy. Thus, deliveries with caesarean section were 41,8% in overweight and obese pregnant women and 58,2% in normal-weight women. Additionally, very high (42.9%), high (45.2%) and medium (41.8%) pregnancy risk was observed for women with a BMI > 25.

**Figure 1:**
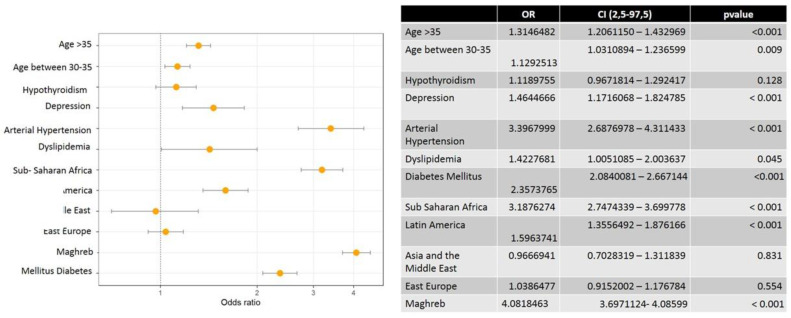
Multivariate analysis of the association of maternal risk factors in women with body mass index (BMI) > 25.

The majority of births were from women from Europe (55.3%) followed by Maghreb (12.9%), Eastern Europe (8.9%), Africa (4.8%), Latin America (4.2%) and Asia (1.2%). Pregnant women from Maghreb, African and Latin American had a significantly high risk of developing obesity. Figure 1 shows the high incidence of overweight and obese pregnant women considering ethnicity. Thus, Maghreb, African and Latin America populations presented OR of 4.08, 3.18 and 1.59, respectively.

The analysis demonstrates significant differences between obese pregnant women and clinical outcomes, such as dyslipidemia, hypertension, diabetes and depression. Thus, 1114 pregnant women presented gestational diabetes mellitus (GDM), of which 30.7% presented overweight and 24.3% obesity. Similarly, 391 pregnant women were diagnosed with hypertension, of which 66.5% had a BMI > 25 (Table 3).

According to the results, hypertension (HT) was the most affected by BMI > 25 (OR = 3.39) followed by gestational diabetes mellitus (GDM) (OR = 2.35) and dyslipidemia and diabetes at OR 1.43 and 1.46, respectively (Figure 1).

## 4. Discussion

This retrospective study is, to the best of our knowledge, the first study that gathered data from pregnant women regarding individual factors during pregnancy and delivery, ethnic information, and metabolic clinical data in our in the Leida region. We analyzed a sample of 16,803 pregnant women, which represents the more than 92% of the total of pregnant women in the health region of Lleida. The prevalence of overweight increased to 4.2% from 2012 to 2018 and obesity in pregnant women was 2.3% from 2012 to 2018. There was an age-related weight gain, we observed a high incidence of overweight and obese pregnant women considering ethnicity, and depression was associated with obesity. According to the results, hypertension (HT) was the most affected by BMI> 25 (OR = 3.39) followed by gestational diabetes mellitus (GDM) (OR = 2.35) and dyslipidemia and diabetes at OR 1.43 and 1.46, respectively.

The incidence rate of overweight and obesity increased from 29.8% in 1980 to 38% in 2013 in middle- and high-income countries, demonstrating an increase in obesity (BMI ≥ 30 kg/m^2^) in women of childbearing age. [12]. The prevalence of obesity in pregnant women was from 11.1% in 2012 to 13.4% in 2018 in our study, which is consistent with the data from European studies. A large retrospective analysis of hospital data from the UK shows that the average prevalence of obesity was 14.6% among women with a single pregnancy [13]. In a study carried out in Finland during 2020, the percentage of overweight women (BMI ≥ 25) obtained before pregnancy was 41.9% and obesity (BMI ≥ 30kg/m^2^) was 17%. Additionally, over two-fifths of Finnish pregnant woman (41.9%) had overweight or obesity (body mass index: 25–29.9 kg/m^2^ and ≥30 kg/m^2^, respectively) in 2019 [14]. Additionally, in a study from the United States (50 US states and the District of Columbia), the decrease in the proportion of normal BMI before pregnancy and the increase in obesity in the years 2013-2018 was observed in the different ethnic groups [15].

In this study of pregnant women (N = 16803), it seems clear that overweight/obesity (N = 5686) are prone to metabolic diseases and delivery complications. Many authors have shown an association between gestational weight gain and adverse maternal and obstetric outcomes [16]. In accordance with our results, pregnant women aged above 30 years, and especially above 35 years, showed higher relations with obese conditions. Although, age < 30 years is thought to present more weight gain during pregnancy [8] other authors describe that women with pregnancy BMI >25 had a higher baseline weight and were more likely to experience excessive gestational weight gain, especially those aged over 30 years [17]. Additionally, the excess weight of pregnant women led to delivery complications such as abortion, preeclampsia and cesarean births. Moreover, in this study, a higher risk (45.25%) of pregnancy was attributed to pregnant women with BMI > 25. Similarly, Goldstein RF et al. demonstrated adverse outcomes of preeclampsia and cesarean delivery [18]. Additionally, Njagu R. et al. found an association between excess weight in pregnant women and neonatal outcomes [4].

Regarding ethnicity, the positive association between Maghreb, African and Latin American pregnant women and BMI > 25 was unexpected. Most likely, the obesogenic environment suggests that an increased availability or access to energy-dense foods that are high in saturated fat and sugar may be related to obesity. Our results are in accordance with Fraser LK et al. who described a relationship between food outlet location, deprivation, weight status and ethnicity [19]. Additionally, Mujahid MS et al. documented in a Californian cohort that racial and ethnic differences, especially for black women, have an impact on maternal [20] morbidity.

Consistent with other large, population-based studies, we noted an association between BMI >25 and clinical outcomes related to metabolic disorders. In particular, HT, GDM and dyslipidemia had significant correlations in pregnant women who were overweight or obese [16]. First, previous retrospective and cross-sectional studies determine that the risk of early-onset and late-onset HT disorders in pregnancy were significantly higher in obese women compared with non-obese women [21]. In this sense, Wagata M et al. constructed a composite variable combining HT in pregnancy and overweight/obesity [22]. The prevalence of hypertensive disorder has increased in the last 10 years, with a 14% prevalence of chronic hypertension during pregnancy and 2–5% gestational hypertension or preeclampsia, which is possibly explained by the corresponding increase in overweight, obesity, diabetes, and maternal age. [23,24]. They observed higher adjusted ORs for HT in overweight/obese women than non-overweight/obese regardless of age. Additionally, Moakye E et al. found a higher baseline prevalence of obesity, age-adjusted influence of HT and preeclampsia among immigrant US populations [25].

In second term, GDM odds ratio demonstrated the positive impact of BMI. Obesity is associated with hyperinsulinemia and insulin resistance, which may be triggered by concomitant low-grade systemic inflammation and subclinical endotoxemia. Accordingly, Forbes S et al. described that disrupted intermediary metabolism contributed to adverse pregnancy outcomes in women with obesity [26]. The authors denoted that obese women have substantial insulin resistance compared with lean women. Consequently, obesity-related pre-pregnancy insulin resistance is associated with a strongly increased risk for GDM [3]. Similarly, other authors noted that women in overweight/obese BMI were more likely to be primiparous and have a lower education level [27]. Nevertheless, women with GDM had an apparent reduction in gestation weight gain when interventions are undertaken. In addition, the effects of inflammatory mediators released by a hypoxic trophoblast together with insulin resistance are the most important factors that determine an adverse effect on pregnancy in patients with GDM or obesity, causing an increased risk of macrosomia, large fetuses, gestational age, shoulder dystocia, and birth trauma [28].

In overweight and obese women, as well as those with excessive weight gain during pregnancy, it is observed a risk for complications during pregnancy, such as GDM, gestational hypertension, preeclampsia, and preterm birth. These conditions place women at an increased risk of future cardiometabolic diseases. A reduction in overweight and obesity, as well as good control of weight gain during pregnancy, could lead to an improvement in associated comorbidities in pregnancy and in the years after delivery [29]. Being overweight or obese before pregnancy (body mass index (BMI) ≥ 25kg m^2^) is the most significant GDM risk factor [30]. The incidence of gestational diabetes mellitus (GDM) is increasing, together with maternal obesity [31]. The European Perinatal Health Report (2010) stated that the proportion of overweight or obese mothers commonly varies from 27% to 37% in European countries [32].

Additionally, an association between hypothyroidism, which includes both subclinical and over hypothyroidism, and risk of GDM, is supported by one study [33], which describes the adequate management of both pathologies considering modifications in lifestyle, such as diet and physical activity.

During pregnancy, total cholesterol levels can reach up to 350 mg/dL, and triglycerides can increase to 300 mg/dL due to increased resistance to insulin, progesterone, estrogens, and placental lactogen. [34]. Singh et al. [35] reported a strong association between dyslipidemia and pregnancy-induced hypertension, as well as intrauterine growth restriction, intrahepatic cholestasis, macrosomia, and fetal death [36]. Thus, the determination of the lipid profile is greatly recommended to introduce rapid management approaches to prevent the damaging effect of dyslipidemia associated with pregnancy [37]. Dyslipidemia has a high global prevalence in all populations (including pregnant women) ranging from 21.7% to 87.7% [38,39,40]. In addition, the most prevalent maternal hypertriglyceridemia (HTG) is due to secondary causes like diabetes and obesity. Obese woman is reported to show net lipolysis at all pregnancy stages. Thus, the developing fetus is exposed to high levels of free fatty acids throughout all stages of in utero development. Therefore, excess lipid and glucose supply to the fetus, in combination with inadequate placental function and in utero environments, are thought to be relevant factors that may also increase the risk of metabolic disease in offspring but also maternal complications such as preeclampsia and preterm labor, among others [41].

The influence of clinical and educational interventions in BMI > 25 women could contribute to improving health, especially for HT, GDM and dyslipidemia disorders. Different studies found that improving interventions in pregnant women might be a solution [42]. Furthermore, evidence in the literature denoted that antenatal diet and physical activity interventions reduced gestational weight gain but with no associated effects on complications [5,43,44,45]. Thus, future decisions to implement behavioral intervention in pregnant women might allow pregnancy complications to be reduced.

In the present study, depression was influenced by BMI > 25. Maternal depression is a serious mental issue that can have a negative impact on the lives of women. Hormonal influence, neurotransmitter function and nutritional deficiencies due to malnutrition or poor nutrition are among the contributing biological factors [46]. In particular, authors described physical activity education, protein, fat, oleic acid, monounsaturated fatty acids, potassium, magnesium and zinc as strong predictors of depression [47]. In accordance with our results, Steining J. et al. concluded that women with obesity are especially vulnerable to antenatal depression [48]. There is a need to develop appropriate screening routines and interventions to mitigate negative health consequences for mothers and offspring. In non-obese pregnant women, lifestyle treatment can reduce depression and body image independently of weight loss, but both lifestyle treatment and weight loss can improve self-esteem [49]. However, Wilson CA et al. found that glycemic load is a key aspect of interventions that aim to optimize the mental health of obese women in the perinatal period [50].

The main strengths of this study included the large sample size and the prospective nature of the clinical data collection. However, this study has several limitations. Firstly, in the data collection, we considered the possible loss of some cases, such as patients who had two gestation periods during the same year where data could not be separated, pregnant women who have been lost when relating pregnancy with the Apgar test, and the weight of the newborns and those of the patients who carry out their follow-up in centers that do not belong to a public health system. It is estimated that prescriptions of this type represent around 2.2% of the total population of the health region; therefore, given the universal coverage of the Spanish National Health System, it is unlikely that they affected the results of the study. Secondly, it is worth to mention that there was no classification of the obesity type in population studied. In the same way, their weight gain data were not collected. Therefore, the present study only considers BMI to elucidate those variables affected by overweight or obesity in pregnant women. Finally, we did not consider the social economic status and education level, and these factors affect maternal obesity, gestational weight gain, and pregnancy outcomes [51].

## 5. Conclusions

Despite these limitations, this analysis provides new insights into the approach of obesity in pregnant women. Overweight and obese pregnant women are at high risk of pregnancy complications. The increasing prevalence of obesity worldwide highlights the importance of our findings and underscores the need for the prevention of metabolic disorders, such as hypertension, diabetes mellitus and dyslidipidemia, especially in pregnant women population. This study documented the influence of BMI for the individual, ethnic and clinical factors of pregnant women. Among individual factors, age (>30 years) is the main factor affected by BMI. Ethnic disparities may affect maternal outcomes, especially for Maghreb, African and Latin America populations. In the present cohort, it is demonstrated that hypertension, diabetes mellitus and dyslidipidemia are highly affected by BMI. In addition, mental health such as depression is aggravated by BMI. It is worth to mentioning that further research on interventions, considering individual, clinical and mental factors of the pregnant women could improve health status of pregnant women. Health policies with cultural interventions for the prevention and control of obesity can improve health conditions in the different ethnic groups of a nation [52,53,54].

## Figures and Tables

**Table 1:** Number of births registered in the Lleida health region by years and number of births in the sample studied with the percentage they represent. Data were collected from IDESCAT.

Year	Deliveries	Sample Deliveries	Sample/Deliveries
2012	3788	2694	71%
2013	3535	2469	70%
2014	3592	2455	68%
2015	3426	2373	69%
2016	3283	2359	72%
2017	3197	2278	71%
2018	3029	2178	72%

**Table 2:** Distribution of body mass index (BMI) in pregnant women.

	<25	26–30	>30
	N = 11,117	N = 3700	N = 1986
Year of delivery			
**2012**	1833 (68.0%)	561 (20.8%)	300 (11.1%)
**2013**	1692 (68.5%)	522 (21.1%)	255 (10.3%)
**2014**	1640 (66.8%)	547 (22.3%)	268 (10.9%)
**2015**	1566 (66.1%)	532 (22.4%)	272 (11.5%)
**2016**	1529 (64.8%)	520 (22.0%)	310 (13.1%)
**2017**	1466 (64.4%)	522 (22.9%)	290 (12.7%)
**2018**	1391 (63.9%)	496 (22.8%)	291 (13.4%)

**Table 3:** Pregnant women characteristics by body mass index (BMI).

	BMI			
	<25	26–30	>30	*p* Value
	N = 11,117	N = 3700	N = 1986	
**Pregnant women age**				<0.001
**<30**	4722 (69.1%)	1381 (20.2%)	734 (10.7%)	
**30–35**	3470 (62.0%)	1392 (24.9%)	739 (13.2%)	
**>35**	2925 (67.0%)	927 (21.2%)	513 (11.8%)	
**Number of pregnancies**				<0.001
**1**	6348 (71.9%)	1645 (18.6%)	838 (9.49%)	
**2**	3237 (63.9%)	1213 (23.9%)	616 (12.2%)	
**3**	1013 (55.4%)	505 (27.6%)	311 (17.0%)	
**4**	325 (52.1%)	189 (30.3%)	110 (17.6%)	
**>4**	194 (42.8%)	148 (32.7%)	111 (24.5%)	
**Preclampsia**				<0.001
**No**	11,037 (66.3%)	3660 (22.0%)	1952 (11.7%)	
**Yes**	80 (51.9%)	40 (26.0%)	34 (22.1%)	
**Multiple pregnancy**				0.474
**No**	11,094 (66.1%)	3696 (22.0%)	1982 (11.8%)	
**Yes**	23 (74.2%)	4 (12.9%)	4 (12.9%)	
**Caesarean section**				<0.001
**No**	9412 (67.8%)	3001 (21.6%)	1461 (10.5%)	
**Yes**	1705 (58.2%)	699 (23.9%)	525 (17.9%)	
**Pregnancy duration**				0.528
**Abortion**	357 (64.4%)	123 (22.2%)	74 (13.4%)	
**Preterm**	218 (67.1%)	69 (21.2%)	38 (11.7%)	
**Term**	479 (63.4%)	176 (23.3%)	101 (13.4%)	
**Prolonged**	7427 (66.5%)	2448 (21.9%)	1296 (11.6%)	
**Risk pregnancy**				<0.001
**No risk**	1566 (54.8%)	718 (25.1%)	573 (20.1%)	
**Medium risk**	2598 (58.2%)	1018 (22.8%)	847 (19.0%)	
**High risk**	177 (57.1%)	73 (23.5%)	60 (19.4%)	
**Very high risk**	5694 (75.7%)	1501 (19.9%)	330 (4.39%)	
**Hypertension**				<0.001
**No**	10,986 (66.9%)	3585 (21.8%)	1841 (11.2%)	
**Yes**	131 (33.5%)	115 (29.4%)	145 (37.1%)	
**Gestational Hypertension**				<0.001
**No**	11,079 (66.4%)	3665 (22.0%)	1939 (11.6%)	
**Yes**	38 (31.7%)	35 (29.2%)	47 (39.2%)	
**Gestational Diabetes Mellitus**				<0.001
**No**	10,616 (67.7%)	3358 (21.4%)	1715 (10.9%)	
**Yes**	501 (45.0%)	342 (30.7%)	271 (24.3%)	
**Dyslidipidemia**				0.027
**No**	11,013 (66.3%)	3652 (22.0%)	1956 (11.8%)	
**Yes**	104 (57.1%)	48 (26.4%)	30 (16.5%)	
**Gestational Dyslidipidemia**				0.833
**No**	11,113 (66.2%)	3699 (22.0%)	1985 (11.8%)	
**Yes**	4 (66.7%)	1 (16.7%)	1 (16.7%)	
**Depression**				0.150
**No**	10,851 (66.3%)	3603 (22.0%)	1924 (11.7%)	
**Yes**	266 (62.6%)	97 (22.8%)	62 (14.6%)	
**Gestational Depression**				0.686
**No**	11,104 (66.2%)	3697 (22.0%)	1983 (11.8%)	
**Yes**	13 (68.4%)	3 (15.8%)	3 (15.8%)	
**Adherence to Diabetes treatment**	2.69 (8.43)	4.35 (12.6)	7.71 (17.5)	<0.001
**Qualitative adherence to diabetes treatment**				0.011
**High**	3 (17.6%)	6 (35.3%)	8 (47.1%)	
**Low**	603 (45.6%)	405 (30.7%)	313 (23.7%)	
**Medium**	1 (16.7%)	1 (16.7%)	4 (66.7%)	
**Diabetes mellitus diagnosis**				<0.001
**No**	10,498 (68.0%)	3279 (21.3%)	1650 (10.7%)	
**Yes**	619 (45.0%)	421 (30.6%)	336 (24.4%)	
**Origin**				<0.001
**Sub-Saharan Africa**	375 (46.0%)	273 (33.5%)	167 (20.5%)	
**Latin America**	454 (63.8%)	176 (24.7%)	82 (11.5%)	
**Asia and Middle East**	157 (73.0%)	45 (20.9%)	13 (6.05%)	
**West Europe**	6782 (73.0%)	1614 (17.4%)	899 (9.67%)	
**East Europe**	1111 (73.6%)	282 (18.7%)	117 (7.75%)	
**Magreb**	893 (40.9%)	848 (38.8%)	442 (20.2%)	

## Data Availability

Available upon request to corresponding author (blanca.salinasroca@udl.cat) or first author (mor14@alumnes.udl.cat).

## References

[1] Weir C.B., Jan A. (2022). BMI Classification Percentile And Cut Off Points. StatPearls.

[2] Drozdz D., Alvarez-Pitti J., Wójcik M., Borghi C., Gabbianelli R., Mazur A., Herceg-Čavrak V., Lopez-Valcarcel B.G., Brzeziński M., Lurbe E. (2021). Obesity and Cardiometabolic Risk Factors: From Childhood to Adulthood. Nutrients.

[3] Reichetzeder C. (2021). Overweight and obesity in pregnancy: Their impact on epigenetics. Eur. J. Clin. Nutr..

[4] Njagu R., Adkins L., Tucker A., Gatta L., Brown H.L., Reiff E., Dotters-Katz S. (2022). Maternal weight gain and neonatal outcomes in women with class III obesity. J. Matern. Neonatal Med..

[5] Gyllensten H., Haby K., Berg M., Premberg Å. Cost effectiveness of a controlled lifestyle intervention for pregnant women with obesity. BMC Pregnancy Childbirth.

[6] Mitchell A., Dunn G.A., Sullivan E.L. (2022). The Influence of Maternal Metabolic State and Nutrition on Offspring Neurobehavioral Development: A Focus on Preclinical Models. Biol. Psychiatry Cogn. Neurosci. Neuroimaging.

[7] Hieronimus B., Ensenauer R. (2021). Influence of maternal and paternal pre-conception overweight/obesity on offspring outcomes and strategies for prevention. Eur. J. Clin. Nutr..

[8] Zhou M., Peng X., Yi H., Tang S., You H. (2022). Determinants of excessive gestational weight gain: A systematic review and meta-analysis. Arch. Public Health.

[9] Ahrens W., Pigeot I., Pohlabeln H., De Henauw S., Lissner L., Molnár D., Moreno L.A., Tornaritis M., Veidebaum T., Siani A. (2014). Prevalence of overweight and obesity in European children below the age of 10. Int. J. Obes..

[10] Siscart J., Orós M., Serna M.C., Perejón D., Galván L., Ortega M. (2022). Adherence to treatment for hypothyroidism in pregnancy and relationship with thyrotropin control: A retrospective observational cohort study. BMC Pregnancy Childbirth.

[11] World Health Organization The SuRF Report 2 Surveillance of Chronic Disease Risk Factors: Country-Level Data and Comparable Estimates The SuRF Report 2 CD-Rom Included The SuRF Report 2-Country-Level Data And Comparable Estimates. http://infobase.who.int.

[12] Ng M., Fleming T., Robinson M., Thomson B., Graetz N., Margono C., Mullany E.C., Biryukov S., Abbafati C., Abera S.F. (2014). Global, regional, and national prevalence of overweight and obesity in children and adults during 1980–2013: A systematic analysis for the Global Burden of Disease Study 2013. Lancet.

[13] Barber C., Rankin J., Heslehurst N. (2017). Maternal body mass index and access to antenatal care: A retrospective analysis of 619,502 births in England. BMC Pregnancy Childbirth.

[14] Zhu Y., Zhang C. (2016). Prevalence of Gestational Diabetes and Risk of Progression to Type 2 Diabetes: A Global Perspective. Curr. Diabetes Rep..

[15] Wang M.C., Freaney P.M., Perak A.M., Greenland P., Lloyd-Jones D.M., Grobman W.A., Khan S.S. (2021). Trends in Prepregnancy Obesity and Association With Adverse Pregnancy Outcomes in the United States, 2013 to 2018. J. Am. Heart Assoc..

[16] Li K., Yang C., Fan J., Li X., Gu C., Liu H. (2022). Prepregnancy body mass index, gestational weight gain, and maternal prepartum inflammation in normal pregnancies: Findings from a Chinese cohort. BMC Pregnancy Childbirth.

[17] Bider-Canfield Z., Martinez M.P., Wang X., Yu W., Bautista M.P., Brookey J., Page K.A., Buchanan T.A., Xiang A.H. (2017). Maternal obesity, gestational diabetes, breastfeeding and childhood overweight at age 2 years. Pediatr. Obes..

[18] Goldstein R.F., Abell S.K., Ranasinha S., Misso M., Boyle J.A., Black M.H., Li N., Hu G., Corrado F., Rode L. (2017). Association of Gestational Weight Gain With Maternal and Infant Outcomes: A Systematic Review and Meta-analysis. JAMA.

[19] Fraser L., Edwards K., Tominitz M., Clarke G., Hill A. (2012). Food outlet availability, deprivation and obesity in a multi-ethnic sample of pregnant women in Bradford, UK. Soc. Sci. Med..

[20] Mujahid M.S., Kan P., Leonard S.A., Hailu E.M., Wall-Wieler E., Abrams B., Main E., Profit J., Carmichael S.L. (2021). Birth hospital and racial and ethnic differences in severe maternal morbidity in the state of California. Am. J. Obstet. Gynecol..

[21] Bicocca M.J., Mendez-Figueroa H., Chauhan S.P., Sibai B.M. (2020). Maternal Obesity and the Risk of Early-Onset and Late-Onset Hypertensive Disorders of Pregnancy. Obstet. Gynecol..

[22] Wagata M., Kogure M., Nakaya N., Tsuchiya N., Nakamura T., Hirata T., Narita A., Metoki H., Ishikuro M., Kikuya M. (2020). Hypertensive disorders of pregnancy, obesity, and hypertension in later life by age group: A cross-sectional analysis. Hypertens. Res..

[23] THL National Institute for Health and Welfare (2018). Medical Birth Register at THL National Institute for Health and Welfare Finland.

[24] Brown M.A., Magee L.A., Kenny L.C., Karumanchi S.A., McCarthy F.P., Saito S., Hall D.R., Warren C.E., Adoyi G., Ishaku S. (2018). Hypertensive Disorders of Pregnancy: ISSHP Classification, Diagnosis, and Management Recommendations for International Practice. Hypertension.

[25] Boakye E., Sharma G., Ogunwole S.M., Zakaria S., Vaught A.J., Kwapong Y.A., Hong X., Ji Y., Mehta L., Creanga A.A. (2021). Relationship of Preeclampsia With Maternal Place of Birth and Duration of Residence Among Non-Hispanic Black Women in the United States. Circ. Cardiovasc. Qual. Outcomes.

[26] Forbes S., Barr S.M., Reynolds R., Semple S., Gray C., Andrew R., Denison F.C., Walker B.R., Norman J. (2015). Convergence in insulin resistance between very severely obese and lean women at the end of pregnancy. Diabetologia.

[27] Hong M., Liang F., Zheng Z., Chen H., Guo Y., Li K., Liu X. (2022). Weight gain rate in the second and third trimesters and fetal growth in women with gestational diabetes mellitus: A retrospective cohort study. BMC Pregnancy Childbirth.

[28] Poblete J.A. (2021). Obesity and Gestational Diabetes in Pregnant Care and Clinical Practice. Curr. Vasc. Pharmacol..

[29] Grieger J.A., Hutchesson M.J., Cooray S.D., Khomami M.B., Zaman S., Segan L., Teede H., Moran L.J. (2021). A review of maternal overweight and obesity and its impact on cardiometabolic outcomes during pregnancy and postpartum. Ther. Adv. Reprod. Health.

[30] Zhang C., Ning Y. (2011). Effect of dietary and lifestyle factors on the risk of gestational diabetes: Review of epidemiologic evidence. Am. J. Clin. Nutr..

[31] Catalano P.M. (2010). Obesity, insulin resistance, and pregnancy outcome. Reproduction.

[32] European Perinatal Health Report (2010). Health and Care of Pregnant Women and Babies in Europe in 2010.

[33] Giannakou K., Evangelou E., Yiallouros P., Christophi C.A., Middleton N., Papatheodorou E., Papatheodorou S.I. (2019). Risk factors for gestational diabetes: An umbrella review of meta-analyses of observational studies. PLoS ONE.

[34] Mauri M., Calmarza P., Ibarretxe D. (2021). Dyslipemias and pregnancy, an update. Clin. Investig. Arterioscler..

[35] Singh A., Kujur A., Jain P. (2018). Feto-maternal impact of altered lipid profile in pregnancy. Int. J. Reprod. Contracept. Obstet. Gynecol..

[36] Wiznitzer A., Mayer A., Novack V., Sheiner E., Gilutz H., Malhotra A., Novack L. (2009). Association of lipid levels during gestation with preeclampsia and gestational diabetes mellitus: A population-based study. Am. J. Obstet. Gynecol..

[37] Pusukuru R. (2016). Evaluation of Lipid Profile in Second and Third Trimester of Pregnancy. J. Clin. Diagn. Res..

[38] Al-Duais M.A., Al-Awthan Y.S. (2019). Prevalence of dyslipidemia among students of a Yemeni University. J. Taibah Univ. Med Sci..

[39] Camacho P.A., Otero J., Pérez M., Arcos E., García H., Narvaez C., Molina D.I., Sanchez G., Duran M., Cure C. (2019). The spectrum of the dyslipidemia in Colombia: The PURE study. Int. J. Cardiol..

[40] Feitosa A.C.R., Barreto L.T., Da Silva I.M., Da Silva F.F., Filho G.S.F. (2017). Impact of the Use of Different Diagnostic Criteria in the Prevalence of Dyslipidemia in Pregnant Women. Arq. Bras. Cardiol..

[41] Li C., Li X., Wu D., Chen Q., Xiao Z., Wen D., Zhai L., Jia L. (2021). Influence of Dietary Behaviors on Dyslipidemia in Pregnant Women and Its Effects on Physical Development of Fetuses and Infants: A Bidirectional Cohort Study. Nutrients.

[42] Al Wattar B.H., Dodds J., Placzek A., Beresford L., Spyreli E., Moore A., Carreras F.J.G., Austin F., Murugesu N., Roseboom T.J. (2019). Mediterranean-style diet in pregnant women with metabolic risk factors (ESTEEM): A pragmatic multicentre randomised trial. PLOS Med..

[43] Greathouse K.L., Padgett R.N., Petrosino J., Hastings-Tolsma M., Faucher M.A. (2022). Exploration of Diet Quality by Obesity Severity in Association with Gestational Weight Gain and Distal Gut Microbiota in Pregnant African American Women: Opportunities for Intervention. Matern. Child Health J..

[44] Wang C., Wei Y., Zhang X., Zhang Y., Xu Q., Sun Y., Su S., Zhang L., Liu C., Feng Y. (2017). A randomized clinical trial of exercise during pregnancy to prevent gestational diabetes mellitus and improve pregnancy outcome in overweight and obese pregnant women. Am. J. Obstet. Gynecol..

[45] Molyneaux E., Begum S., Briley A.L., Seed P.T., Howard L.M., Poston L., on behalf of the UPBEAT consortium (2018). Do elevated symptoms of depression predict adherence and outcomes in the UPBEAT randomised controlled trial of a lifestyle intervention for obese pregnant women?. BMC Pregnancy Childbirth.

[46] Salehi-Pourmehr H., Dolatkhah N., Gassab-Abdollahi N., Farrin N., Mojtahedi M., Farshbaf-Khalili A. (2019). Screening of depression in overweight and obese pregnant women and its predictors. J. Obstet. Gynaecol. Res..

[47] Blau L.E., Hormes J.M. (2020). Preventing Excess Gestational Weight Gain and Obesity in Pregnancy: The Potential of Targeting Psychological Mechanisms. Curr. Obes. Rep..

[48] Steinig J., Nagl M., Linde K., Zietlow G., Kersting A. (2017). Antenatal and postnatal depression in women with obesity: A systematic review. Arch. Women’s Ment. Health.

[49] Jiskoot G., De Loos A.D., Beerthuizen A., Timman R., Busschbach J., Laven J. (2020). Long-term effects of a three-component lifestyle intervention on emotional well-being in women with Polycystic Ovary Syndrome (PCOS): A secondary analysis of a randomized controlled trial. PLoS ONE.

[50] Wilson C.A., Seed P., Flynn A.C., Howard L.M., Molyneaux E., Sigurdardottir J., Poston L. (2020). Is There an Association Between Diet, Physical Activity and Depressive Symptoms in the Perinatal Period? An Analysis of the UPBEAT Cohort of Obese Pregnant Women. Matern. Child Health J..

[51] Huynh M., Borrell L.N., Chambers E.C. (2014). Maternal Education and Excessive Gestational Weight Gain in New York City, 1999–2001: The Effect of Race/Ethnicity and Neighborhood Socioeconomic Status. Matern. Child Health J..

[52] Singh G.K., Siahpush M., Hiatt R.A., Timsina L.R. (2011). Dramatic Increases in Obesity and Overweight Prevalence and Body Mass Index Among Ethnic-Immigrant and Social Class Groups in the United States, 1976–2008. J. Community Health.

[53] Singh G.K., Lin S.C. (2013). Dramatic Increases in Obesity and Overweight Prevalence among Asian Subgroups in the United States, 1992–2011. ISRN Prev. Med..

[54] Siega-Riz A.M. (2012). Prepregnancy Obesity: Determinants, Consequences, and Solutions. Adv. Nutr. Int. Rev. J..

